# Differences in Prenatal Tobacco Exposure Patterns among 13 Race/Ethnic Groups in California

**DOI:** 10.3390/ijerph16030458

**Published:** 2019-02-05

**Authors:** Sumi Hoshiko, Michelle Pearl, Juan Yang, Kenneth M. Aldous, April Roeseler, Martha E. Dominguez, Daniel Smith, Gerald N. DeLorenze, Martin Kharrazi

**Affiliations:** 1 Environmental Health Investigations Branch, California Department of Public Health (CDPH), Richmond, CA 94804, USA; michelle.pearl@cdph.ca.gov (M.P.); danlfsmith@gmail.com (D.S.); marty.kharrazi@cdph.ca.gov (M.K.); 2 Sequoia Foundation, La Jolla, CA 92037, USA; juan.yang@cdph.ca.gov (J.Y.); gndel@yahoo.com (G.N.D.); 3 Wadsworth Center, Division of Environmental Health Sciences, New York State Department of Health, Albany, NY 12201, USA; kenneth.aldous@health.ny.gov; 4 California Tobacco Control Program, CDPH, Sacramento, CA 95814, USA; april.roeseler@cdph.ca.gov; 5 Fusion Center, CDPH, Sacramento, CA 95814, USA; martha.dominguez@cdph.ca.gov

**Keywords:** tobacco, smoking, cotinine, prenatal, environmental tobacco smoke, passive smoking, Native American, Asian, race, ethnicity

## Abstract

Prenatal tobacco exposure is a significant, preventable cause of childhood morbidity, yet little is known about exposure risks for many race/ethnic subpopulations. We studied active smoking and environmental tobacco smoke (ETS) exposure in a population-based cohort of 13 racially/ethnically diverse pregnant women: white, African American, Hispanic, Native American, including nine Asian/Pacific Islander subgroups: Chinese, Japanese, Korean, Filipino, Cambodian, Vietnamese, Laotian, Samoan, and Asian Indians (N = 3329). Using the major nicotine metabolite, cotinine, as an objective biomarker, we analyzed mid-pregnancy serum from prenatal screening banked in 1999–2002 from Southern California in an effort to understand differences in tobacco exposure patterns by race/ethnicity, as well as provide a baseline for future work to assess secular changes and longer-term health outcomes. Prevalence of active smoking (based on age- and race-specific cotinine cutpoints) was highest among African American, Samoan, Native Americans and whites (6.8–14.1%); and lowest among Filipinos, Chinese, Vietnamese and Asian Indians (0.3–1.0%). ETS exposure among non-smokers was highest among African Americans and Samoans, followed by Cambodians, Native Americans, Vietnamese and Koreans, and lowest among Filipinos, Japanese, whites, and Chinese. At least 75% of women had detectable cotinine. While for most groups, levels of active smoking corresponded with levels of ETS, divergent patterns were also found. For example, smoking prevalence among white women was among the highest, but the group’s ETS exposure was low among non-smokers; while Vietnamese women were unlikely to be active smokers, they experienced relatively high ETS exposure. Knowledge of race/ethnic differences may be useful in assessing disparities in health outcomes and creating successful tobacco interventions.

## 1. Introduction

Tobacco exposure remains the leading preventable cause of death and disease in the U.S. [1]. Prenatal tobacco exposure, through active smoking or environmental tobacco smoke (ETS), is one of the most significant avoidable causes of maternal and child morbidity and mortality [1,2]. Deleterious effects include pre-term birth, fetal growth restriction, low birth weight, miscarriage, specific congenital malformations, childhood leukemia, reduced maternal fertility, adverse respiratory, neurological and cardiovascular effects, and infant death [2,3,4,5,6,7,8,9,10]. Compared to active smoking, the effects of ETS are similar and consistent though attenuated [11,12,13,14]. The effects of prenatal exposure may manifest in childhood, with increased risk of impaired lung function, asthma, obesity, cognitive deficits and detrimental cardiovascular consequences [15,16,17,18,19,20,21,22,23]. Epigenetic changes from in-utero exposure to tobacco have been detected in children [24], and prenatal smoking has been shown to influence smoking habits of future generations [25,26].

Despite over 50 years of tobacco control policies that have reduced overall self-reported smoking prevalence in the U.S., smoking prevalence during pregnancy in the U.S. declined only slightly in the decade between 2000 to 2010 from 13.3% in 2000 to 12.3%, making the U.S. Healthy People 2020 goal of 1.4% unlikely in the near future [3]. Furthermore, the true smoking prevalence is likely higher; a validation study that measured serum cotinine, a biomarker for nicotine, estimated that 23% of pregnant and 9% of non-pregnant female smokers did not disclose their smoking [27]. Data on exposure to ETS is not routinely collected, and self-report is poorly correlated with objective biomarkers [28,29].

Without consistent, sensitive and objective measures, it is unclear what the true exposure differences between race/ethnic subgroups of pregnant women are [1,3,30], which is critical in designing effective, targeted intervention strategies and ultimately reducing disparities. National surveillance data based on self-report showed variability in prenatal smoke exposure patterns between different race/ethnic groups, with the highest rates of prenatal smoking in American Indian/Alaskan Native women, followed by white and African American women, and lower rates in Hispanics and Asians [31]. Using broad race/ethnic categories can obscure differences between subgroups [32], and federal data collection standards for race categories have been found to mask heterogeneity in smoking patterns [33]. Although Asians are the fastest growing race/ethnic group in the U.S. [34], studies of smoking patterns have categorized Asian/Pacific Islander as a single group or more typically been limited to white, African-American and sometimes Hispanic groups. Even less is known about racial/ethnic variability in ETS exposure, which may not follow patterns for active smoking due to gender and cultural differences in active smoking [35]. Accurate understanding of race/ethnic tobacco exposure rates is further complicated by differences in women’s non-disclosure patterns by race/ethnicity [27], and patterns of quitting smoking during pregnancy, which also vary by race/ethnic group [3].

Using cotinine measurements from prenatal serum and associated demographic data, our aim was to describe active smoking and ETS exposure during pregnancy by race/ethnicity in a multi-ethnic population. Subjects from a population-based cohort of pregnant women in Southern California in 1999–2002 were selected to represent 13 racially/ethnically diverse populations: white, African American, Hispanic, Native American, and nine understudied Asian/Pacific Islander subgroups: Chinese, Japanese, Korean, Filipino, Cambodian, Vietnamese, Laotian, Samoan, and Asian Indian. We describe race/ethnic tobacco exposure patterns among these diverse groups, providing a baseline for future work to assess secular changes in exposure patterns and association with long-term disease outcomes. We chose the major nicotine metabolite, cotinine, as a biomarker and a highly sensitive laboratory method in order to obtain an objective measure of exposure from active smoking and ETS exposure within these different groups.

## 2. Materials and Methods

### 2.1. Study Population

We sampled pregnant women from San Diego, Orange, and Imperial counties who participated in California’s statewide prenatal screening program between November 1999 and December 2002. During this period, California offered prenatal screening to pregnant women in mid-pregnancy to detect several chromosomal and other congenital disorders [36]. Typically, a single blood specimen was collected from the enrollee between 15 and 20 weeks of pregnancy, and banked as part of Project Baby’s Breath (PBB), a population-based research study in the 2000s established by several investigators of the present study (Kharrazi, DeLorenze and Pearl) [37]. PBB created a biological specimen bank and database based on probabilistically-linked birth certificate, prenatal screening and other records. It was designed to enable cross-sectional and longitudinal investigations concerning environmental exposures and periconception, pregnancy, and subsequent health outcomes. Funding for cotinine analysis of race/ethnicity subgroups within PBB was obtained in 2005. From the 191,441 prenatal screening records corresponding to banked PBB prenatal specimens, 300 subjects (except where noted) were randomly selected, from each of the following Asian/Pacific Islander race/ethnic groups: Asian Indian, Cambodian, Chinese, Filipino, Japanese, Laotian, Korean, Samoan (N = 184), and Vietnamese. Subjects from four other race/ethnic groups with available specimens were also included: Hispanic (N = 97), white (N = 103) and African American (N = 300) and Native American (N = 245); (total N with specimens = 3329). Demographic variables other than race/ethnicity were based on linked birth record data (mother’s age; years of education; public versus private payment source for birth or delivery services; and foreign- versus U.S.-born status). Of the 3329 records with specimens, 3054 linked with birth record data, and another 40 had missing values for one or more demographic characteristics, resulting in 3014 subjects with complete data (both serum cotinine measurements and demographic data).

Screening program enrollees signed consent forms allowing for research and confidential use of program data and specimens by the State of California or State-approved scientific researchers without identifying the person or persons from whom these results were obtained, unless an enrollee specifically prohibited such use in writing. Women who requested that their specimens not be used for research, which was rare, were not included in the study. Approval for the research project was obtained from the California Health and Human Services Agency Committee for the Protection of Human Subjects in 2003 (#13-02-1072).

### 2.2. Laboratory Cotinine Analysis

PBB contracted with the regional newborn and prenatal screening laboratory serving San Diego, Orange, and Imperial counties during November 1999 to December 2002 to bank blood specimens. Initially, serum specimens were retained in original serum separator tubes at −20 °C with accompanying blood cell pellets. Between June 2000 and December 2002, serum was aliquotted at the regional laboratory within 1–2 days of testing into a single 4 mL sterile polypropylene non-pyrogenic, cryogenic vial and stored at −20 °C. Serum cotinine levels were measured by the Wadsworth Center, Division of Environmental Disease Prevention, New York State Department of Health, from November 2007 to June 2008 using a highly sensitive isotope dilution high performance liquid chromatography/tandem mass spectrometry (LC/MS/MS) method. The analysis was a modification of techniques used by the Centers for Disease Control and Prevention [38]. A small subset (N = 27 Hispanic and N = 34 white) were analyzed for cotinine using the same liquid chromatographic-atmospheric pressure chemical ionization tandem mass spectrometry method by the Tobacco Exposure Biomarkers Section, Division of Laboratory Sciences, Centers for Disease Control and Prevention in 2006. For validation purposes, a subset of serum specimens from PBB were tested by both laboratories (N = 28). Correlation between the two laboratory values, calculated as raw cotinine minus overall average of study blanks, was 0.99. The method had a quantitation limit of 0.025 ng/mL and a limit of detection of 0.001 ng/mL.

### 2.3. Data Analysis

Demographic and socioeconomic characteristics were examined by race/ethnicity among records with complete data (N = 3104). In the total population (N = 3329), prevalence of active smoking among the different race/ethnic groups was assessed based on measured serum cotinine cutpoints as recommended by Benowitz et al. [39] as follows: For adults (age > 19 years), 5 ng/mL for non-Hispanic whites, 6 ng/mL for non-Hispanic blacks, and 1 ng/mL for Mexican-Americans; for adolescents (age 12–19 years), 3 ng/mL for non-Hispanic whites and blacks, and 1 ng/mL for Mexican-Americans. The recommended cutpoint of 3 ng/mL for the overall U.S. population was used for subjects not falling in the above categories.

Women with serum cotinine values below these cutpoints were designated as nonsmokers in their respective age/race/ethnic groups (N = 3180). All serum cotinine values in ng/mL were initially rounded to three decimal places, and values below the detection limit (0.001 ng/mL) were included after assigning the value of 0.001/√2. We used values below the quantitation limit (0.025 ng/mL) and above the detection limit to most closely estimate the lognormal distribution of exposure in study subjects. Prevalence of detectable ETS exposure among non-smokers was assessed (serum cotinine > 0.001 and less than the designated cutpoint) for each race/ethnic group, as well as the distribution of and geometric means of cotinine values (with 95% confidence intervals) and interquartile ranges. Tests to determine differences in mean log10 serum cotinine between race/ethnic groups were performed using the SAS ANOVA procedure and the Least Significant Difference (LSD) approach to account for multiple comparisons (SAS Statistical Software, v9.4, SAS Institute, Cary, NC, USA).

Among non-smokers, multivariable linear regression of log serum cotinine was used to evaluate whether the crude relationships between race/ethnicity were due to other demographic factors. Age was modeled as a continuous and a quadratic term, while education (less than 16 years versus more) and insurance (publicly funded/indigent versus private) were modeled as dichotomous covariates. Indicator variables were included for each race/ethnic group (except white women) in order to compare mean log cotinine for each group relative to white women after adjusting for other demographic factors. Race/ethnicity and other demographic variables were evaluated both individually and as groups of variables.

## 3. Results

All demographic characteristics evaluated varied widely by race/ethnicity (Table 1, dataset with complete demographic values, N = 3014). For example, 4.4% of mothers of Chinese background were age 25 or younger; and at the other end of the spectrum, 47.5% of African American mothers fell in this age category. The percent of Chinese mothers with less than 16 years of education was 20.4%, compared to 91.8% of Samoans. Chinese mothers had the lowest percent of public funding (3.3%), followed by Japanese (7.2%); with Hispanic mothers at 62.2%. The group with the highest percent of foreign-born mothers were Vietnamese (98.6%); the Asian/Pacific Islander subgroup with the lowest percentage of foreign-born mothers was Samoan (42.4%).

Prevalence of active smoking, as determined by measured cotinine levels, varied between 0.3 and 14.1% (Table 2; dataset with specimens, N = 3329). The race/ethnic groups with the highest percentages of active smokers (in decreasing order) were Samoan, African American, Native American and white (6.8–14.1%). Although numbers were small and thus statistical distinctions between groups uncertain, Korean, Cambodian, Hispanic, Japanese and Laotian race/ethnic groups appeared to have intermediate levels of active smoking (2.0–4.0% prevalence), and the groups with the lowest prevalence included women of Filipino, Chinese, Vietnamese and Asian Indian background (0.3–1.0%).

Of our total population of 3329 with measured cotinine levels, we identified 3180 women as non-smoking. Prevalence of ETS exposure as measured by geometric mean cotinine among non-smoking women was highest among the following groups: African Americans and Samoans, followed by Cambodians, Native American, Vietnamese and Koreans (Table 3). The groups with the lowest prevalence of ETS exposure were Filipino, Japanese, white, and Chinese. These race/ethnic rankings tracked similarly with percent of detectable cotinine, and the vast majority of women had detectable cotinine levels (75.0–90.5%).

**Figure 1:**
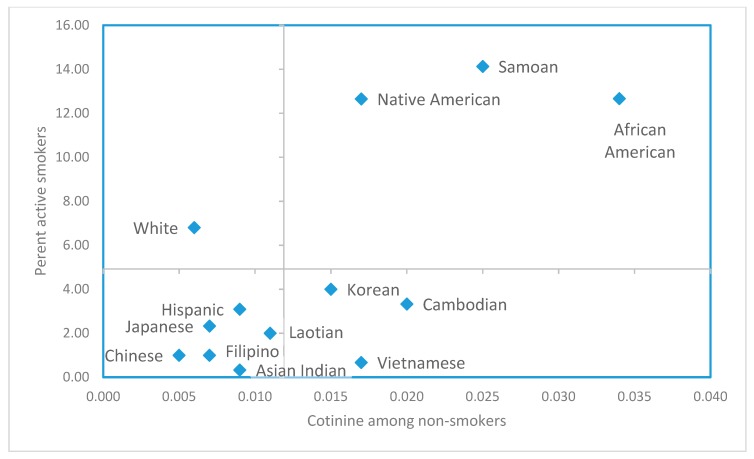
Percent active smokers (N = 3329); vs geometric mean serum cotinine levels in non-smokers (N = 3180); prenatal screening program enrollees in 13 race/ethnic groups; San Diego, Orange and Imperial counties. (Smoking status defined by race- and age-specific cutpoints per Benowitz et al. 2008 [39]. Grid lines show the mean of percent active smokers in the 13 race/ethnic groups and the antilog of the mean log cotinine in non-smokers in the 13 groups.)

Comparison of active smoking and ETS exposure patterns are visualized in Figure 1, which shows prevalence of active smoking for each race/ethnic group along with each group’s geometric mean cotinine level among non-smokers. While consistent patterns were apparent for many groups, e.g., high active smoking prevalence and high ETS exposure among non-smokers, divergent patterns were suggested in other groups. The upper right quadrant represents race/ethnic groups with both high active smoking and high ETS exposure (African Americans, Samoans, and Native Americans). However, while smoking prevalence among white women was relatively high among these race/ethnic groups, the average level of ETS exposure was low among non-smoking white women (upper left quadrant). While prevalence of active smoking among Vietnamese women was low, their ETS exposure was relatively high. Cambodians and Koreans also fell in this quadrant, but most Asian groups clustered in the lower left quadrant, suggesting low levels of both active smoking and ETS exposure. Samoans were an exception, standing out from other Asian/Pacific Islander groups with both high levels of active smoking and ETS exposure, similar to Native Americans and African Americans. Hispanics appeared to have more similar smoking patterns to the Asian groups in the lower left quadrant, characterized by low active smoking and low/intermediate ETS exposure.

Race/ethnicity, as a group including all race/ethnic subtypes, was strongly associated with serum cotinine level (log10 serum cotinine, ng/mL) in multivariable regression among non-smokers (*p* < 0.001) (Table 4). Using both linear and quadratic age variables in the model improved the fit over using the linear term alone, based on Akaike Information Criterion (AIC) values, so both terms were included in the final model. All demographic variables (excluding race/ethnicity), separately and as a group, were also significantly associated with cotinine concentration (*p* ≤ 0.001, grouped demographic variables). After adjusting for these variables, race/ethnic differences persisted although there was some shifting of ETS rankings compared to unadjusted results in Table 3, with Koreans ranking highest and Hispanics lowest. Being born outside the U.S. was associated with lower cotinine, but was not significantly associated with cotinine concentration in multivariate models with demographic and race/ethnicity variables and so was not retained in the final model (data not shown).

## 4. Discussion

Using serum cotinine as an objective biomarker for tobacco smoke exposure over the previous few days in mid-pregnancy, our study identified significantly different exposure prevalence for active smoking and ETS exposure among 13 different race/ethnic populations, suggesting that lifestyle and social patterns associated with tobacco exposure differ between groups. Among non-smokers, most pregnant women were exposed to ETS. As expected, socioeconomic factors and maternal age were clearly important predictors of ETS exposure. Race/ethnicity remained strongly associated even after these adjustments. Based on the unadjusted results, which inform public health practice, the subpopulations with high active smoking and ETS exposure included African American, Samoan and Native American women. ETS exposure was also elevated among Cambodians, Vietnamese and Koreans, and lowest among Chinese, white, Japanese, Filipino, Hispanic, and Asian Indian women. Active smoking and ETS exposure patterns did not always track together across groups; for example, white women had high active smoking and low ETS exposure, while Korean, Cambodian and Vietnamese women had moderate to low active smoke exposure, but high ETS exposure.

The varying rates of active smoking and ETS exposure across race/ethnic groups may relate to many factors, including language barriers, lack of insurance, barriers to participation in smoking cessation programs and other cultural and socioeconomic factors. The discordant active smoking and ETS exposure patterns between different race/ethnic groups seen in Figure 1 may be a reflection of gender differences in tobacco use or social stratification. Smoking behavior is a cultural norm in some communities, such as among men in Southeast Asian countries, and smoking prevalence remains high among males in those race/ethnic groups in the U.S. [40]. A study of non-smoking Vietnamese adults in California found high secondhand exposure in home environments for women [41]. Social dynamics may play a role; a study of family communication in Vietnamese and Chinese families identified a pattern of “suffering in silence [42].” In a study of Asian/Pacific Islanders in Hawaii, native Hawaiians with large social networks were found to have lower smoking rates, but this was not so for Filipinos or East Asians [43]. The East Asian category, however, was comprised of Chinese, Japanese and Koreans. In our analysis, pregnant nonsmokers who were Chinese had relatively low exposure to ETS, but Koreans were among the groups with higher ETS exposure.

Race/ethnic factors beyond family and social dynamics may affect smoking patterns as well, such as the context of residential enclaves and segregation. Living in a Mexican residential enclave is associated with decreased smoking during pregnancy for mothers of Mexican origin, while living in a non-Hispanic white neighborhood was associated with increased smoking [44]. Residence in a less racially segregated area has been related to a lower risk of smoking during pregnancy among African American women, but higher smoking risk among Asian and Hispanic women [45]. African-Americans and other minorities may also encounter more tobacco advertising and greater access to tobacco products in their communities [46,47].

In addition to race/ethnic differences in smoking rates, cessation-related knowledge and practices may differ as well. Native Hawaiians and Filipinos were found in a previous study to have lower knowledge and use of cessation methods and products than Caucasians, suggesting that these groups may be underserved [48]. Tobacco companies have specifically targeted Asian immigrants and Asian Americans [49], while few interventions have been directed towards these communities [32]. To address this gap, the Centers for Disease Control and Prevention’s “Racial and Ethnic Approaches to Community Health” (REACH) project was launched to reduce racial/ethnic community health disparities using community-driven anti-smoking strategies [40]. Knowledge of differences in smoking patterns for specific ethnic groups is critical, as culturally appropriate interventions have been found to be more effective [32,50,51,52].

Tobacco control policies have been demonstrated to reduce prenatal smoking rates [53,54,55]. Decreases in adverse maternal and child outcomes have also been demonstrated to be affected by tobacco control policies [56,57]. The introduction of increased cigarette taxes and marketing bans have shown a 5–10% reduction in adverse maternal and child outcomes [53]. The effectiveness of tobacco control policies may also vary by race/ethnic group, possibly modified by socioeconomic status. A study of the impact of increased cigarette taxes found that smoking rates were most improved among white and African American mothers with the least education [55]. However, investigators also found that increased taxes were associated with a slight increase in the number of cigarettes smoked per month among Hispanic mothers without a high school degree and Asians with a college degree or higher.

Pregnant women represent a key nexus in the effort to control the nation’s tobacco exposure. From the direct effects on the fetus that may manifest in poor birth outcomes, to those that develop in childhood and later in the lifespan, to parental smoking and the child’s ETS exposure, and continuing to intergenerational and epigenetic effects on descendants, pregnancy presents an opportunity to intervene in the trajectory of the most significant preventable cause of death and disease in the U.S. Strategies are needed that acknowledge the role of both parents and family members or others living in the home. Also of critical importance is addressing the upcoming generation of future mothers and fathers who are currently children and adolescents in order to interrupt smoking initiation and lifelong addiction. Unfortunately, progress in reducing tobacco exposure has been hindered by rising e-cigarette use, which has tripled among high school and middle school children between 2013 and 2014, surpassing traditional cigarette use for the first time, according to the National Youth Tobacco Survey [58].

To our knowledge, this is the first study to biochemically quantify exposure to tobacco in this variety of ethnic subgroups, particularly for Asian/Pacific Islander subpopulations. The study is population-based, deriving from all prenatal screening program participants in the southern part of the state. The cotinine assay is the most sensitive available, allowing quantification at low levels of ETS. The period of study, 1999–2002, predated the introduction of e-cigarettes, so the serum cotinine levels measured here were largely through exposure to nicotine in tobacco smoke.

The study has several limitations. Despite our efforts to sample understudied race/ethnic populations, subject numbers were smaller than ideal, especially for analysis of populations with lower prevalence of active smoking. However, our ETS analyses are more robust. Given that the half-life of cotinine is shorter in pregnancy (around 9 h versus 17 h in non-pregnant [59]), and the use of a single specimen to measure serum cotinine in this study, there is opportunity to misclassify true smokers as non-smokers, especially light or intermittent smokers, or women who quit before the second trimester. This same problem could raise cotinine levels in non-smoker groups, but given use of the geometric mean, a measure of the central tendency for a log normal distribution, this metric should be minimally impacted. Although we used the most valid cutpoints as based on race and age-specific values and appropriate to the time period of our study, these values were only available for three race/ethnic groups. However, a sensitivity analysis using a serum cotinine cutpoint of 10 ng/mL to define smoking for all race/ethnic groups did not reveal any material differences in the results presented. No cutpoints have been defined specifically for pregnant women, despite differences in metabolism that could affect their cotinine levels [59]. Additionally, the timing of the prenatal screening, which occurs between 15 and 20 weeks of pregnancy, may not reflect exposure during other critical exposure windows of time and could miss women who had smoked but had quit earlier in pregnancy. The study will also miss women who do not participate in prenatal screening. Previous research has found that women under 20 or over 35 years of age, with late or no prenatal care, or public insurance, are less likely to participate in prenatal screening [60], although it is uncertain how these selection factors may influence the distribution of active smoking and ETS exposure. It is possible that the tobacco exposure patterns we identified were influenced by factors beyond those considered in this examination, including potential differences across the groups in use of certain medications, alcohol consumption, and genetic factors affecting the metabolism of nicotine and cotinine [61]. It is unknown whether the combination of these unmeasured factors would change the conclusions of this paper.

## 5. Conclusions

Substantial variability in patterns of active smoking and ETS exposure during pregnancy was revealed among the 13 race/ethnic groups in the present study. Prevalence of active smoking was highest among African American, Samoan, Native American and white women, and lowest among Asian Indians, Vietnamese, Chinese, and Filipinos. Among non-smokers, ETS exposure was highest among African Americans and Samoans, followed by Cambodians, Native Americans, Vietnamese and Koreans, and lowest among Filipino, Japanese, white, and Chinese women. While high active smoking was often accompanied by high ETS exposures, discordant patterns included white women with relatively high rates of active smoking, but low ETS exposure among non-smokers, and relatively high ETS exposure among Vietnamese women although they rarely smoked.

While cultural differences may likely have persisted since the early 2000s when this study’s blood specimens were collected, follow-up surveillance that distinguishes specific subgroups is needed to assess current exposure levels. These historical exposure differences may lead to subsequent differences in pediatric outcomes and even beyond into adulthood. Comprehensive tobacco control and prevention programs have been effective in reducing tobacco use in the U.S., and in particular in California [62,63]. However, the disparities in active tobacco use and ETS exposure among race/ethnic groups underscore the importance of culturally and ethnically relevant interventions and surveillance, including verification based on biospecimen testing for accuracy, in order to advance progress towards the goal of reducing tobacco’s harm.

## Figures and Tables

**Table 1:** Demographic characteristics of prenatal screening program enrollees in 13 race/ethnic groups, San Diego, Orange and Imperial counties (N = 3014) *.

Race/Ethnicity	N = 3014	Column %	Mother’s Age ≤ 25 Years (%)	Mother’s Education < 16 Years (%)	Mother’s Insurance Public Funding (%)	Mother is Foreign-Born (%)
White	95	3.2	20.0	49.5	14.7	13.7
African American	263	8.7	47.5	87.5	57.0	8.7
Hispanic	82	2.7	37.8	89.0	62.2	70.7
Asian Indian	274	9.1	11.3	29.2	11.3	91.6
Cambodian	274	9.1	43.1	82.8	41.2	90.9
Chinese	275	9.1	4.4	20.4	3.3	88.7
Filipino	274	9.1	17.9	51.8	14.2	76.6
Japanese	265	8.8	5.7	42.6	7.2	65.3
Korean	262	8.7	6.1	29.8	17.2	94.3
Laotian	280	9.3	32.9	81.1	32.9	90.4
Native American	213	7.1	40.4	85.9	33.8	8.0
Samoan	170	5.6	41.8	91.8	42.9	42.4
Vietnamese	287	9.5	13.2	74.9	31.4	98.6
Chi Square *p*-value			<0.0001	<0.0001	<0.0001	<0.0001

* Of 3329 total records with serum cotinine specimens, 3014 linked with birth records and had complete data for demographic variables.

**Table 2:** Smoking prevalence (95% confidence interval) during mid-pregnancy for prenatal screening program enrollees in 13 race/ethnicity groups; San Diego, Orange and Imperial counties; by descending prevalence (N = 3329).

Race-Ethnicity	No	No of Smokers *	%	95% Confidence Interval
Samoan	184	26	14.1	9.8–19.9
African American	300	38	12.7	9.4–16.9
Native American	245	31	12.7	9.1–17.4
White	103	7	6.8	3.3–13.4
Korean	300	12	4.0	2.3–6.9
Cambodian	300	10	3.3	1.8–6.0
Hispanic	97	3	3.1	1.1–8.7
Japanese	300	7	2.3	1.1–4.7
Laotian	300	6	2.0	0.9–4.3
Filipino	300	3	1.0	0.3–2.9
Chinese	300	3	1.0	0.3–2.9
Vietnamese	300	2	0.7	0.2–2.4
Asian Indian	300	1	0.3	0.1–1.9

* Smoking status defined by race- and age-specific cutpoints per Benowitz et al. 2008 [39].

**Table 3:** Comparisons of mid-pregnancy serum cotinine levels between 13 race/ethnic groups of non-smoking * prenatal screening program enrollees; mean geometric serum cotinine levels with 95% confidence intervals; percent detectable serum cotinine; and interquartile ranges; San Diego, Orange and Imperial counties (N = 3180).

Race-Ethnicity ^†^	N	% Detectable Cotinine	Geometric Mean Cotinine (ng/mL) ^‡^	95% Confidence Interval	IQR 25–75th Percentile
African American ^a^	262	90.5	0.034	0.026–0.044	0.009–0.151
Samoan ^a,b^	158	88.0	0.025	0.018–0.034	0.010–0.077
Cambodian ^b,c^	290	90.3	0.020	0.016–0.025	0.006–0.066
Native American ^c^	214	84.6	0.017	0.013–0.022	0.004–0.058
Vietnamese ^c^	298	90.3	0.017	0.014–0.020	0.006–0.046
Korean ^c,d^	288	88.2	0.015	0.012–0.018	0.006–0.039
Laotian ^d,e^	294	80.6	0.011	0.009–0.014	0.003–0.039
Asian Indian ^e,f^	299	82.3	0.009	0.008–0.011	0.003–0.030
Hispanic ^e,f,g^	94	79.8	0.009	0.006–0.012	0.003–0.028
Filipino ^f,g^	297	75.1	0.007	0.006–0.009	0.001–0.030
Japanese ^f,g,h^	293	75.4	0.007	0.005–0.008	0.001–0.020
White ^g,h^	96	75.0	0.006	0.004–0.009	0.001–0.019
Chinese ^h^	297	75.4	0.005	0.004–0.006	0.001–0.017

* Non-smoking status defined by race- and age-specific cutpoints per Benowitz et al. 2008 [39]. ^†^ Lettered superscripts indicate no statistically significant differences between race/ethnic groups in geometric mean serum cotinine levels (*p* < 0.05). For example, no distinction can be made between African Americans and Samoans as to which group had higher environmental tobacco smoke (ETS) exposure, and African Americans had higher ETS exposure than any other race/ethnic group other than possibly Samoans. ^‡^ By descending geometric mean cotinine level.

**Table 4:** Regression analysis of race/ethnicity and other demographic characteristics and environmental tobacco smoke (ETS) exposure (log_10_ serum cotinine, ng/mL) among non-smoking * prenatal screening program enrollees; San Diego, Orange and Imperial counties (N = 2880) ^†^.

Variable	Parameter Estimate (log_10_ Cotinine, ng/mL) ^‡^	Pr > F
Model R^2^ = 0.18		
Intercept	−0.687	0.044
Demographic covariates (as group)		<0.0001
Mother has less than 16 years of education	0.233	<0.0001
Mother’s care is publicly funded	0.346	<0.0001
Mother’s age (continuous)	−0.098	<0.0001
Mother’s age (squared term)	0.001	0.001
Race/ethnic covariates * (as group)		<0.0001
Korean	0.429	<0.0001
African-American	0.403	<0.0001
Samoan	0.367	<0.0002
Vietnamese	0.319	0.0004
Cambodian	0.269	0.0030
Native American	0.228	0.0165
Asian Indian	0.223	0.0125
Japanese	0.120	0.1825
Laotian	0.108	0.2298
Chinese	0.084	0.3472
Filipino	0.050	0.5727
Hispanic	−0.207	0.0716

* Non-smoking status defined by race- and age-specific cutpoints per Benowitz et al. 2008 [39]. ^†^ Subjects with complete demographic data. ^‡^ Difference in mean log_10_ cotinine concentrations for each race/ethnic group, white as reference; race/ethnic groups displayed in descending order of magnitude of parameter estimate.
